# Awareness of HIV functional cure and willingness in participating in related clinical trials: comparison between antiretroviral naïve and experienced men who have sex with men living with HIV

**DOI:** 10.1186/s12879-022-07346-x

**Published:** 2022-04-15

**Authors:** Tsz Ho Kwan, Chin Pok Chan, Ngai Sze Wong, Shui Shan Lee

**Affiliations:** 1 Stanley Ho Centre for Emerging Infectious Diseases, The Chinese University of Hong Kong, Shatin, Hong Kong; 2 Jockey Club School of Public Health and Primary Care, The Chinese University of Hong Kong, Shatin, Hong Kong

**Keywords:** HIV, Functional cure, Men who have sex with men, Awareness

## Abstract

**Background:**

Human immunodeficiency virus (HIV) functional cure is a novel biomedical strategy characterized by sustained viral suppression without the need for life-long medications. The attitude of people living with HIV (PLHIV) towards functional cure and clinical trials are understudied. We aimed to examine the awareness and levels of anticipation for HIV functional cure among men who have sex with men (MSM) living with HIV, and their willingness to join trials as differentiated by their antiretroviral treatment status.

**Methods:**

MSM living with HIV with and those without treatment history were recruited from Hong Kong’s HIV specialist clinics. Self-administered questionnaires covering behavioral profile, perceived impact of HIV cure, attitude towards HIV functional cure and related clinical trials were collected. Clinical data were separately transcribed. Determinants of perceptions and attitudes were identified by logistic regression models.

**Results:**

Of 356 MSM living with HIV recruited, less than half (42%) were aware of HIV functional cure, but they had a high level of anticipation for it. Treatment-experienced participants were more likely to be aware of HIV functional cure. Awareness was associated with continued engagement in sexual activities after HIV diagnosis and sexually transmitted infection (STI) diagnosis. Higher anticipation was observed among older MSM living with HIV but it was negatively associated with one’s awareness. Over 90% were willing to join functional cure trials, especially those who had previously been diagnosed with STI and had engaged in chemsex in the past year. Advice from healthcare professional was an important factor considered by those willing to join clinical trials. Younger, better educated MSM, and those with lower CD4 counts were more concerned about potential risk of AIDS and potential complications upon trial participation.

**Conclusions:**

MSM living with HIV, especially those sexually active, showed positive attitude towards functional cure and willingness to join related clinical trials despite low awareness. To enhance preparedness for HIV functional cure trials, community education, updated information and appropriate medical advice would be needed. Safety is a major concern for potential enrollees in HIV functional cure trials.

**Supplementary information:**

The online version contains supplementary material available at 10.1186/s12879-022-07346-x.

## Background

Life-long treatment with antiretroviral compounds is currently the gold standard in the clinical management of human immunodeficiency virus (HIV) infection. While antiretrovirtal therapy is effective in restoring health and minimizing secondary HIV transmission, it falls short of achieving virus eradication. Effective curative treatment could provide a sustainable solution to prevention and control [1]. Despite increasing research with the accumulation of scientific evidence for HIV cure, such therapy is not yet in sight [2]. Despite the uncertainties, a survey of over 400 people living with HIV (PLHIV) in the United States showed that more than half were willing to participate in different HIV cure studies [3]. Elimination of the HIV virus in the “Berlin patient” offered new hope in the PLHIV communities about the ultimate goal of cure [4]. De-stigmatization was particularly valued by PLHIV who favored curative treatment when it becomes available [5, 6].

In the interim, animal studies and human trials suggested that “functional cure” of HIV infection may become a reality in the coming years [7, 8]. Unlike “sterilizing cure”, functional cure strategy aims to achieve effective suppression of HIV viral load so that antiretroviral therapy (ART) becomes unnecessary. While “functional cure” could represent different strategies, the term is now consistently used in referring to the attainment of virus control without ART [7], while one’s HIV infection status remains unchanged. Willingness of PLHIV in receiving non-eradication cure treatment or joining functional cure research would be an important consideration, especially that current generations of antiretrovirals are safe and extremely effective. PLHIV’s decision may hinge on one’s understanding of the concept of cure and how this is explained [9]. In the United States, many participants in a study did not consider functional cure as an improvement to conventional ART [5]. Experiences with ART may also affect PLHIV’s decision about participation in functional cure research. In Hong Kong, ART coverage in PLHIV receiving care at the public service is high. With the increasing reports of the promising outcome of functional cure, it is timely that their attitude towards participation in functional cure research be explored. We hypothesized that the degree of knowledge and attitudes towards HIV cure and functional cure differed between ART-experienced and ART-naïve PLHIV. In addition, education needs in the specific area of functional cure among newly diagnosed patients could be identified, which may in turn ease subject recruitment for a clinical trial, and improve expectation management of participants. As such, we undertook to examine the attitude of MSM living with HIV towards functional cure in Hong Kong, and contrast the awareness and perceptions between newly diagnosed and veteran PLHIV who have been on long duration of ART.

## Methods

Data for analyses came from a questionnaire survey administered on ART-naïve and ART-experienced men who have sex with men (MSM) living with HIV participating in two separate cohort studies in Hong Kong. The former study comprised the collection of data from newly diagnosed MSM attending any one of the three HIV specialist clinics over a two-year period; while the latter study involved treatment-experienced MSM receiving ART at the largest HIV clinic during the same period of time as a follow-up round of a study on the sex networking behaviors following HIV diagnosis [10]. Informed consents were obtained prior to the conduct of the surveys. Approval of the Joint Chinese University of Hong Kong-New Territories East Cluster Clinical Research Ethics Committee and Ethics Committee of Department of Health were obtained.

A self-administered questionnaire was designed with mutual topics in both surveys covering (a) demographics and HIV diagnosis year, (b) perceptions about HIV cure and their impacts, and (c) awareness and anticipation of HIV functional cure, considerations of and concerns about participating in an HIV functional cure clinical trial (Additional file 1). Functional cure was the specific theme for this survey, the definition of which was clearly described in order to avoid the misinterpretation of the results as there could be confusion between different forms of HIV curative treatment. Hypothesizing that sexually active PLHIV would have a higher awareness, anticipation and interest in HIV functional cure, sexual behaviors in the preceding year including sexually transmitted infection (STI) diagnosis, sexual activity, and use of psychotropic drugs for sex (chemsex), were inquired. Clinical data were separately transcribed, which included the age of HIV diagnosis, acquired immunodeficiency syndrome (AIDS) status, and longitudinal CD4 cell count and viral load. The most recent readings prior to the time of questionnaire for the latter two measures were recorded for analyses.

On the perceived impacts of HIV cure, participants were asked to choose from seven options: no longer needing to take HIV medications, restoration and stabilization of effective immune function, not getting HIV for a second time, no longer needing to visit a doctor for HIV, no longer at risk of AIDS or HIV-related morbidity, no longer transmitting HIV to the others, and being considered as a person not living with HIV. Awareness of HIV functional cure was categorized into: knew of it and understood what it is, heard of the idea but not the details, and had no idea of it. After giving a brief introduction on HIV functional cure, participants were asked to rate their anticipation on a scale of 0 to 10. Willingness to join an HIV functional cure study was assessed by a 6-point Likert scale (definitely no, probably no, maybe no, maybe yes, probably yes, and definitely yes). Participants were inquired about the importance of each of the following factors which they would consider in joining an HIV functional cure trial: safety, duration of the clinical trial, incentives for participation, view and support from family and peers, advice from healthcare professionals, credibility of the research institution, and need for interruption of ART medications. Potential adverse consequences of participating in the trial included CD4 count going down, HIV viral load going up, becoming infectious to the others, presence of AIDS or other related complications, and side effects arising from the therapy. The levels of importance and concern were ranked in descending order as: very important/concerned, moderately important/concerned, somewhat important/concerned, and a minimally important/concerned.

Differences in perceptions on HIV cure and HIV functional cure trial between ART-naïve and experienced groups were assessed by participants’ sociodemographic and behavioral factors in logistic regression. Determinants of anticipation of HIV cure, awareness of HIV functional cure, and willingness to join an HIV functional cure trial were analyzed by univariable and multivariable logistic regression models. Participants were defined as having a high degree of anticipation if they gave a score of 10. Awareness of HIV functional cure was reflected by at least knowing the idea without details. The importance of factors associated with joining functional cure trials and the concerns were dichotomized by defining the lower two levels (“a little important” and “somewhat important”, and “a little concerned” and “somewhat concerned”) as zero and the rest (“moderately important” and “very important”, and “moderately concerned” and “very concerned”) as one. Only variables with p < 0.10 were included in the initial multivariable models for stepwise backward elimination based on the AIC value. Inter-correlations between cure- and trial-related consideration factors and concerns were explored using logistic regression models. All statistical analyses were conducted in R.

## Results

### Demographics

Between March 2019 and January 2020, 153 treatment-naïve MSM were recruited, which accounted for about one-third of all newly diagnosed male PLHIV in Hong Kong; while data from 203 treatment-experienced MSM were collected between April 2019 and November 2020. The median age of MSM included in the analyses was 37 years (interquartile range [IQR] 29–47 years), with treatment-naïve ones at 30 years (IQR 26–38 years) and treatment-experienced ones at 42 years (IQR 35–50 years) of age (Table 1). Among those receiving treatment, the median duration of HIV diagnosis and receiving ART was 9 years (IQR: 7–13 years) and 8 years (IQR: 6–12 years), respectively. The median age of HIV diagnosis of all enrolled MSM was 31 years (IQR 26–38 years). As regards sexual behaviors, about one-third (38%) did not have any sexual activities in the preceding year, while 39% had engaged in chemsex, among whom 12% injected drugs. About 90% of treatment-experienced participants have achieved a viral load below 20 copies/mL, while almost all treatment-naïve counterparts gave a value of at least 1000 copies/mL. Regarding CD4 cell count, a smaller proportion (16%) of treatment-naïve participants had at least 500 cells/uL, whereas three-quarters in the treatment-experienced group had attained the same level.

**Table 1:** Participants’ socio-demographics, sexual and clinical characteristics by treatment status

	Treatment-naïve (n = 153)		Treatment-experienced (n = 203)		Total^a^ (N = 356)	
	n	%	n	%	n	%
Age group (median = 37, IQR = 29–47)						
16–29	74	48.4	23	11.5	97	27.5
30–39	47	30.7	60	30.0	107	30.3
40–49	17	11.1	59	29.5	76	21.5
50 or over	15	9.8	58	29.0	73	20.7
Education level						
Secondary or below	50	33.6	65	32.0	115	32.3
Post-secondary or above	103	67.3	138	68.0	241	67.7
Employment status						
Full-time/self-employed	109	71.2	148	77.9	257	74.9
Part-time/freelancer	14	9.2	120	10.5	34	9.9
Student	12	7.8	0	0	12	3.5
Others	18	11.7	22	11.6	40	11.7
STI history in the past year						
No	116	75.8	136	68.3	252	71.6
Yes	37	24.2	63	31.7	100	28.4
Sexual activity after HIV diagnosis						
No	103	67.3	31	15.6	134	38.1
Yes	50	32.7	168	84.4	218	61.9
Engagement in chemsex in the past year						
No	76	49.7	116	60.7	192	55.8
Yes	77	50.3	75	39.3	152	44.2
Age at HIV diagnosis (median = 31, IQR = 26–38)						
16–29	78	51.0	74	37.0	152	43.1
30–39	43	28.1	79	39.5	122	34.6
40–49	17	11.1	39	19.5	56	15.9
50 or over	15	9.8	8	4.0	23	6.5
History of AIDS						
No	142	92.8	176	86.7	318	89.3
Yes	11	7.2	27	13.3	38	10.7
CD4 cell count (cells/uL)						
≥ 500	25	16.3	158	77.8	183	51.4
< 500	128	83.7	45	22.2	173	48.6
HIV viral load (copies/mL)						
< 20	2	1.3	180	89.6	182	51.4
20–999	5	3.3	18	9.0	23	6.5
1000–99,999	71	46.4	2	1.0	73	20.6
≥ 100,000	75	49.0	1	0.5	76	21.5

*STI* sexually transmitted infections, *HIV* human immunodeficiency virus, *MSM* men who have sex with men, *AIDS* acquired immunodeficiency syndrome

^a^Total number for each variable may not add up to N = 356 due to missing data

### Perceived benefits of HIV cure

Perceptions on HIV cure differed between the treatment-experienced and naïve group (Table 2). The most commonly perceived impact of HIV cure was “restoration and stabilization of effective immune function” (63%), with lower odds among those on ART compared to ART-naïve participants (odds ratio [OR] 0.36, 95% confidence interval [CI] 0.23–0.58). About half (56%) considered untransmittability as an important impact of curing HIV infection. Lowered risk of AIDS or related morbidity was considered more important in the treatment-naïve group (46% vs. 35%, OR 0.62, 95% CI 0.40–0.95), whereas reduced need for clinic visit was accorded higher importance among treatment-experienced participants (18% vs. 34%, OR 2.28, 95% CI 1.38–3.78). Those who considered immune function stabilization as an important impact of HIV cure had a lower CD4 cell count (OR 3.49, 95% CI 2.20–5.52) and were less likely to have recently been engaging in sex (OR 0.30, 95% CI 0.19–0.50) (Table 3). Perceived importance of reduction of clinic visits was associated with being sexually active (OR 1.85, 95% CI 1.11–3.08) and a higher CD4 level (OR 1.86, 95% CI 1.15–3.00). Participants having attained post-secondary education level (OR 1.75 95% CI 1.09–2.81) and younger than 40 years of age regarded being free from the risk of AIDS or HIV-related morbidity as an important impact for HIV cure. Older adults (those aged 50 years or above) did not consider reduced transmittability important (OR 0.23, 95% CI 0.12–0.43). De-labelling was an important impact for those with a higher education level (OR 1.73, 95% CI 1.00-2.97).

**Table 2:** Participants’ (a) perceptions of the impacts of HIV cure, (b) perception and attitudes towards HIV functional cure, and (c) consideration about HIV functional cure trial, by treatment status

	Treatment-naïve (n = 153) (reference)		Treatment-experienced (n = 203)			
	n	%	n	%	OR (95% CI)	
*(a) Perceived impacts of HIV cure*						
No longer needing to take HIV medications	62	40.5	97	48.3	1.37 (0.89–2.09)	
Restoration and stabilization of effective immune function	116	75.8	107	53.2	0.36 (0.23–0.58)***	
Not getting HIV for a second time	20	13.1	33	16.4	1.31 (0.72–2.38)	
No longer needing to visit a doctor for HIV	28	18.3	68	33.8	2.28 (1.38–3.78)**	
No longer at risk of AIDS or HIV-related morbidity	71	46.4	70	34.8	0.62 (0.40–0.95)*	
No longer be transmitting HIV to others	95	62.1	105	52.2	0.67 (0.44–1.02)	
Being considered as a person not living with HIV	34	22.2	59	29.4	1.45 (0.89–2.37)	
*(b) Perception and attitude towards HIV functional cure*						
Awareness of HIV functional cure						
Never heard about it	94	62.3	109	54.0	1.00	
Heard but didn’t know the details	21	13.9	71	35.1	2.92 (1.67–5.10)***	
Heard and understood what it is	36	23.8	22	10.9	0.53 (0.29–0.96)*	
Level of anticipation for HIV functional cure						
< 10	59	38.6	78	40.0	0.94 (0.61–1.45)	
10	94	61.4	117	60.0	1.00	
Willingness in joining a functional cure trial						
Maybe/probably/definitely no	10	6.5	16	7.9	0.82 (0.36–1.85)	
Maybe/probably/definitely yes	143	93.5	187	92.1	1.00	
*(c) Considerations about HIV functional cure trial*						
Important factors to be considered about the trial						
Safety of the therapy	148	96.7	195	97.0	1.10 (0.33–3.67)	
Duration of the clinical trial	123	80.4	159	79.1	0.92 (0.55–1.56)	
Incentives for participation	40	26.1	40	20.3	0.72 (0.44–1.19)	
Views and support from family and peers	50	32.7	51	26.0	0.72 (0.46–1.15)	
Advice from healthcare professionals	139	174	90.8	87.0	0.67 (0.34–1.34)	
Credibility of the research institution	132	86.3	180	89.6	1.36 (0.72–2.60)	
Interruption of HIV antiretroviral medications	135	88.2	166	83.4	0.67 (0.36–1.24)	
Concerns about consequences of joining a trial						
CD4 count going down	118	77.1	143	71.9	0.76 (0.47–1.23)	
HIV viral load going up	132	86.3	163	81.5	0.70 (0.39–1.26)	
Becoming infectious to the others	123	80.4	147	73.9	0.69 (0.41–1.15)	
Presence of AIDS or other related complications	140	91.5	166	83.0	0.45 (0.23–0.89)*	
Adverse effects of therapy	127	83.0	161	80.5	0.85 (0.49–1.46)	

HIV human immunodeficiency virus; AIDS acquired immunodeficiency syndrome

*p < 0.05 ** p < 0.01 *** p < 0.001

**Table 3:** Crude odds ratio for impacts of perceived importance for HIV cure among MSM (N = 356)

	Impacts of perceived importance for HIV cure						
	IMP1^a^	IMP2^a^	IMP3^a^	IMP4^a^	IMP5^a^	IMP6^a^	IMP7^a^
Age group							
16–29	1.00	1.00	1.00	1.00	1.00	1.00	1.00
30–39	0.85 (0.48–1.48)	0.65 (0.36–1.15)	1.18 (0.56–2.48)	1.21 (0.64–2.30)	0.58 (0.33–1.02)	0.60 (0.34–1.06)	1.80 (0.97–3.35)
40–49	1.46 (0.80–2.66)	0.68 (0.36–1.27)	0.83 (0.35–1.96)	1.39 (0.70–2.76)	0.54 (0.29-1.00)*	0.90 (0.48–1.71)	0.98 (0.48–2.02)
50 or over	1.27 0.69–2.35)	0.98 (0.51–1.89)	0.69 (0.28–1.74)	1.74 (0.88–3.45)	0.50 (0.27–0.94)*	0.23 (0.12–0.43)***	1.07 (0.52–2.21)
Education level							
Secondary or below	1.00	1.00	1.00	1.00	1.00	1.00	1.00
Post-secondary or above	1.51 (0.96–2.38)	1.01 (0.64–1.60)	0.61 (0.34–1.11)	1.70 (1.00-2.9)	1.75 (1.09–2.81)*	0.65 (0.41–1.02)	1.73 (1.00-2.97)*
STI history in the past year	1.16 (0.73–1.85)	0.76 (0.47–1.23)	1.24 (0.66–2.33)	1.33 (0.80–2.22)	1.22 (0.76–1.95)	1.44 (0.89–2.32)	0.90 (0.52–1.54)
Sexual activity after HIV diagnosis	1.07 (0.69–1.64)	0.30 (0.19–0.50)***	0.95 (0.52–1.75)	1.85 (1.11–3.08)*	0.94 (0.61–1.46)	0.86 (0.56–1.33)	1.55 (0.93–2.58)
Engagement in chemsex	1.03 (0.67–1.58)	0.71 (0.46–1.11)	0.88 (0.48–1.64)	0.95 (0.58–1.54)	1.24 (0.80–1.91)	1.05 (0.68–1.62)	0.90 (0.55–1.46)
CD4 cell count (cells/uL)							
≥ 500	1.00	1.00	1.00	1.00	1.00	1.00	1.00
< 500	0.79 (0.52–1.20)	3.49 (2.20–5.52)***	0.78 (0.43–1.41)	0.54 (0.33–0.87)*	1.24 (0.81–1.89)	1.04 (0.68–1.58)	1.05 (0.65–1.68)
About Functional cure							
Anticipation	2.15 (1.38–3.36)***	1.56 (1.00-2.43)	0.87 (0.48–1.58)	1.08 (0.66–1.76)	1.34 (0.86–2.08)	0.61 (0.39–0.94)*	0.96 (0.59–1.58)
Awareness	1.04 (0.68–1.60)	0.60 (0.39–0.93)*	1.19 (0.66–2.16)	1.01 (0.63–1.63)	0.85 (0.55–1.31)	0.73 (0.48–1.12)	1.31 (0.81–2.11)
Willingness	0.68 (0.30–1.51)	2.10 (0.94–4.70)	0.97 (0.32–2.92)	1.01 (0.41–2.49)	1.87 (0.77–4.58)	2.20 (0.97-5.00)	0.79 (0.33–1.88)

*MSM* men who have sex with men, *HIV* human immunodeficiency virus, *STI* sexually transmitted infections, *AIDS* acquired immunodeficiency syndrome

^a^IMP1 No longer need to take HIV medications; IMP2 Restoration and stabilization of effective immune function; IMP3 Not getting HIV for a second time; IMP4 No longer need to visit a doctor for HIV; IMP5 No longer at risk of AIDS or HIV-related morbidity; IMP6 No longer transmitting HIV to the others; IMP7 Being considered as a person not infected with HIV

* p < 0.05 ** p < 0.01 *** p < 0.001

### Awareness of HIV functional cure

Overall, less than half (42%) of the participants were aware of HIV functional cure. Compared with those having no knowledge of it, treatment-experienced MSM were more likely to have heard of HIV functional cure but without detailed knowledge (adjusted odds ratio [aOR] 2.92, 95% CI 1.67–5.10). Awareness was associated with STI history in the past year (aOR 1.64, 95% CI 1.00-2.68), and being sexually active (aOR 1.90, 95% CI 1.14–3.18) (Table 4). Those who were aware of HIV functional cure did not consider restoration and stabilization of effective immune function an important impact (OR 0.60, 95% CI 0.39–0.93), nor were they concerned about potential adverse effects of therapies for functional cure (OR 0.54, 95% CI 0.31–0.93).

**Table 4:** Crude and adjusted odds ratio for awareness and anticipation of HIV functional cure and willingness in participating in a trial among MSM (N = 356)

	Awareness		Level of anticipation		Willingness to participate in functional cure trial	
	Crude OR	Adjusted OR	Crude OR	Adjusted OR	Crude OR	Adjusted OR
Age group						
16–29	1.00	–	1.00	1.00	1.00	–
30–39	1.01 (0.58–1.76)	–	0.87 (0.50–1.52)	0.88 (0.50–1.56)	0.72 (0.25–2.10)	–
40–49	0.74 (0.40–1.36)	–	1.28 (0.69–2.37)	1.18 (0.62–2.22)	1.19 (0.32–4.37)	–
50 or over	0.98 (0.53–1.82)	–	2.34 (1.18–4.61)*	2.55 (1.28–5.11)**	0.62 (0.20–1.94)	–
Education level						
Secondary or below	1.00	–	1.00	–	1.00	–
Post-secondary or above	0.97 (0.62–1.52)	–	1.10 (0.69–1.74)	–	1.12 (0.48–2.59)	–
STI history in the past year	1.54 (0.97–2.46)	1.64 (1.00-2.68)*	1.23 (0.75–1.99)	1.46 (0.88–2.42)	9.94 (1.32–74.65)*	8.16 (1.06–62.77)*
Sexual activity after HIV diagnosis	1.60 (1.03–2.50)*	1.90 (1.14–3.18)*	1.08 (0.70–1.69)	–	1.84 (0.82–4.17)	2.11 (0.87–5.13)
Engagement in chemsex	0.82 (0.53–1.27)	–	1.25 (0.80–1.94)	–	3.42 (1.25–9.33)*	2.35 (0.83–6.61)
CD4 cell count (cells/uL)						
≥ 500	1.00	1.00	1.00	–	1.00	–
< 500	1.22 (0.80–1.87)	1.59 (0.97–2.61)	0.89 (0.58–1.37)	–	0.80 (0.36–1.78)	–
About functional cure						
Awareness	**–**	**–**	0.48 (0.31–0.75)**	0.47 (0.30–0.74)**	0.85 (0.38–1.90)	0.52 (0.21–1.25)
Anticipation	0.48 (0.31–0.75)**	0.46 (0.29–0.72)***	**–**	**–**	1.74 (0.77–3.93)	–
Willingness to participate in trial	0.85 (0.38–1.90)	–	1.74 (0.77–3.93)	–	–	–

*MSM* men who have sex with men, *HIV* human immunodeficiency virus, *STI* sexually transmitted infections, *OR* odds ratio

* p < 0.05 ** p < 0.01 *** p < 0.001

### Anticipation of HIV functional cure

Among all participants, the median anticipation score for HIV functional cure was 10 (IQR 8–10) with more than half (59%) giving a score of 10, and it did not differ between ART treatment-naïve and experienced participants. Among treatment-experienced MSM, those having been diagnosed with HIV for more than 8 years had higher odds of scoring 10 for their level of anticipation for HIV cure (OR 1.93, 95% CI 1.08–3.46). Those anticipating HIV functional cure were more likely to be of age 50 years or above (aOR 2.55, 95% CI 1.28–5.11), and to have a lower awareness of the idea (aOR 0.47, 95% CI 0.30–0.74) (Table 4). Higher level of anticipation was associated with the consideration of not needing to take long-term HIV medications following functional cure (OR 2.15, 95% CI 1.38–3.36) but negatively with untransmittability of HIV (OR 0.61, 95% CI 0.39–0.94).

### Acceptance of an HIV functional cure trial

Should there be an HIV functional cure trial, the vast majority (93%) would consider joining. Therapy safety (96%), advice from healthcare professionals (88%), and credibility of the research institution (88%) were the top three factors participants took into account when considering whether to join such a trial. Participants interested in joining the trial were more likely to have been diagnosed with an STI in the past year (aOR 8.16, 95% CI 1.06–62.77), and to take advice from healthcare professionals when making such a decision (OR 4.64, 95% CI 1.84–11.69). When deciding whether to participate in an HIV functional cure trial, view and support from family and peers were important for those who ceased to have sex (OR 1.99, 95% CI 1.18–3.34) and who had attained lower education level (OR 2.31, 95% CI 1.42–3.74) (Additional file 2: Table S1). Credibility of the research institution was important to those who had received higher education (OR 2.11, 95% CI 1.10–4.04). Participating in such a trial is not without concerns. Major concerns included the potential risk of developing AIDS and complications (86%), HIV viral load going up (83%) and adverse effects of the therapy (81%). They were concerned about adverse effects on their CD4 level (OR 1.84, 95% CI 1.09–3.10) and complications (OR 2.16, 95% CI 1.06–4.42) after joining the trial. Treatment-experienced participants were less likely to be concerned about AIDS or complications (OR 0.45, 95% CI 0.23–0.89), whereas participants aged below 30 years (OR 4.76, 95% CI 1.66–13.64), those who had attained post-secondary level education (OR 2.08, 95% CI 1.11–3.88), and who had a CD4 cell count lower than 500 cells/uL (OR 2.02, 95% CI 1.06–3.84), expressed concerns about the risk of developing AIDS or related complications after or during trial participation.

Concerns arising from participating in an HIV functional cure study were intertwined with factors that were considered when deciding on whether one would join such a study. Those concerned about interruption of ART regimen were worried about CD4 count going down (OR 2.30, 95% CI 1.24–4.27), HIV viral load going up (OR 2.81, 95% CI 1.43–5.52) and becoming infectious (OR 2.01, 95% CI 1.06–3.80) (Additional file 2: Table S2) following functional cure therapy. Considering trial duration important was associated with concerns about adverse effects (OR 2.67, 95% CI 1.47–4.84). Participants who considered trial safety important were concerned about all five potential adverse consequences of participating in the trial. Those looking forward to the prospect of restoring effective immunity following HIV cure had concerns about CD4 count going down after joining the trial (OR 2.03, 95% CI 1.25–3.29).

## Discussion

HIV functional cure is a relatively new concept for PLHIV. Despite their generally low awareness, MSM living with HIV had, as shown in this study, a high level of anticipation for HIV functional cure, especially those who had been diagnosed and put on ART for some years. The effective achievement of functional cure carries the potential prospect of getting rid of repeated clinic visits and taking long-term medications [11]. The benefit of being untransmittable may, however, not be regarded as an important impact for HIV cure because in the current era of “undetectable equals untransmittable”, adherence to ART is already effective in minimizing the risk of transmission. In our cohorts of MSM living with HIV, the attitudes differed by one’s ART treatment experience. Treatment-naïve participants were more concerned about their immune function and AIDS progression than those ART-experienced, as deteriorated health was perceived by newly diagnosed patients an imminent consequence brought on by HIV [12]. Anticipation for HIV functional cure was apparently lower among those who were aware of it. Although some 42% had heard about functional cure, most had limited knowledge of it and cared little about the potential benefit of restoring their immune function and potential adverse effects arising from the new therapy. With the increasing likelihood that functional cure may soon become the next major advance in HIV treatment [7], there is the need for promoting community education on the rationale and strategy for HIV functional cure. As in many countries, MSM accounted for a high proportion of PLHIV in Hong Kong [13], and who continued to be significantly impacted by the epidemic [14]. The MSM community, especially those living with HIV would need to be targeted in anticipation of the introduction of functional cure therapy. In our study, MSM engaging in higher risk sexual behaviors were more likely to be aware of the recent advancement on HIV science, such as pre-exposure prophylaxis [15]. This echoed our results that sexually active PLHIV, particularly those engaged in condomless sex as indicated by their STI history, were more likely to be aware of HIV functional cure.

There are diverse reasons for PLHIV to look forward to receiving functional cure therapy. It is intuitive that PLHIV with a weaker immune system were less likely to be sexually active and they wished to restore and stabilize their immune function and were at the same time more concerned about adverse HIV outcomes after joining functional cure trials. The fact that younger PLHIV cared more about disease development and transmittability was likely because they were newly diagnosed and would remain sexually active for a longer time in the future. PLHIV with higher education level regarded de-labelling of HIV an important impact of HIV cure. In Hong Kong, public stigma against PLHIV [16] and perceived discrimination [17] have been reported. It is therefore an ideal vision for them to remove the label of HIV after achieving some sort of cure. Discrimination in workplace and difficulty in career development could be experienced by PLHIV [18]. Although highly educated PLHIV were found to deal better with workplace discrimination and improve employment quality than those with lower education level [19], they were more concerned with their career development that removing stigma means removing a barrier in their career paths. From the perspective of the general public, increased awareness and knowledge in HIV science advancement might be able to reduce stigma. A previous study showed that heterosexually active adults who were aware of the untransmittable nature of PLHIV achieving undetectable viral load had a lower level of anticipated HIV stigma [20]. As this study focuses on PLHIV, a follow-up study in the general population and the MSM community would be needed to assess the impact of functional cure on general, and dating- and sex-related enacted stigmas.

Overall, the idea of an HIV functional cure trial was well accepted by our cohorts of MSM living with HIV with over 90% indicating their willingness to join, similar to the high acceptance rate of 95% in a previous study [11]. Potential trial participants were more likely to have engaged in higher risk sexual activities as reflected by their report of STI in the preceding year. This might be precipitated by a prospect of continuation or re-engagement in adventurous condomless behaviors upon cure [21]. Safety is a major concern for clinical trials involving the use of novel agents like neutralizing antibodies or therapeutic vaccines. Careful monitoring of PLHIV in the trial would be warranted not just for protecting participants’ health but also easing their concerns of side effects and adverse HIV outcomes. Hesitation would arise if the trial requires interruption of ART or if it takes a long time, in relation to the risk of rebound and potential adverse effects [11]. Trusting relationship between PLHIV, researchers and healthcare workers could be beneficial for ensuring the delivery of optimal health outcomes [22]. Based on potential participants’ trust on healthcare professional advice, collaboration with HIV clinics on explaining the details and safety issues of the trial would be paramount.

This study carries some limitations. Like other epidemiological studies, there were inherent recall and social desirability biases in the administration of behavioral questionnaires. We had minimized these biases by adopting a shorter recall period and omitting fine details about sexual activities. The adoption of a self-administering approach should have reduced embarrassments in response to interviewers’ questions. Incorporation of clinical data by transcription benefited the study by providing objective measurements of participants’ HIV outcomes. In the absence of any functional cure trials, the exploration of one’s willingness to participate in a hypothetical trial without having one in place could be postulational. Nevertheless, the systematic inquiries into the awareness and anticipation of functional HIV cure among MSM living with HIV and their inclination in joining a hypothetical study have generated useful results which could be of reference for the planning of a functional cure trial.

## Conclusions

To PLHIV, HIV cure means restoring and stabilizing their immune systems that they no longer need to take long-term medications, nor do they need to visit HIV clinics repeatedly. While virus eradication cannot be achieved, HIV functional cure is a promising strategy in which MSM living with HIV had a high level of anticipation despite relatively low awareness. While a high acceptance rate of a functional cure clinical trial was elicited, MSM living with HIV were concerned about adverse HIV outcomes. Their appreciation of the objectives of functional cure, understanding of the study procedures, and recognition of potential adverse events are crucial, and all of which need to be accessible well before a functional cure trial begins enrolment.

## Supplementary Information

**Additional file 1.** Questionnaire.

**Additional file 2:  Table S1.** Crude odds ratio for factors of importance to be considered and concerns related to participation in functional cure trial among MSM (N=356). **Table S2.** Crude odds ratio for important factors to be considered about and concerns related to the trial among MSM (N=356).

## Data Availability

The datasets generated during and/or analysed during the current study are not publicly available due to ethical considerations but are available from the corresponding author on reasonable request and approval from the ethics committees.

## Abbreviations

HIV: Human immunodeficiency virus
PLHIV: People living with HIV
MSM: Men who have sex with men
STI: Sexually transmitted infection
AIDS: Acquired immunodeficiency syndrome
IQR: Inter-quartile range
OR: Odds ratio
aOR: Adjusted odds ratio
ART: Antiretroviral therapy
