# Supplementary material for: Awareness of HIV functional cure and willingness in participating in related clinical trials: comparison between antiretroviral naïve and experienced men who have sex with men living with HIV
Source: BMC Infect Dis. 2022 Apr 15;22:383. doi: 10.1186/s12879-022-07346-x (PMC9013029; doi:10.1186/s12879-022-07346-x)
Supplement: Supplementary file 1 — Additional file 1. Questionnaire. [file 12879_2022_7346_MOESM1_ESM.docx]

# Treatment-naïve participants

1. What is your year of birth? __________________

2. What is your highest level of education?

- Primary or below
- Secondary
- Diploma or associate degree
- Degree or above

3. What is your employment status?

- Full-time work
- Part-time work / Freelance only
- Student
- Housewife
- Unemployed
- Retired
- Others (Please specify): __________

4. In the 1-year period before infection, were you diagnosed with any other sexually transmitted infections?

- Yes
- No

5. After diagnosis, which type of male sex partners were you inclined to have sex with?

- I definitely looked for HIV-ve sex partner(s).
- I was inclined to find HIV-ve sex partner(s).
- I was inclined to find HIV+ve sex partner(s).
- I definitely looked for HIV+ve sex partner(s).
- I did not care about the HIV status of my sex partner/s.

6. After diagnosis, did you take entertainment drugs before having sex with another man?

- No
- Yes, but less often than before diagnosis
- Yes, with similar frequency as before diagnosis
- Yes, and more often than before diagnosis

7. In your opinion, what are the most important impacts of an HIV cure? (Choose 3 options at most).

- No longer at risk of AIDS or HIV-related morbidity
- No longer need to take HIV medications
- Restoration and stabilization of effective immune function
- No longer transmitting HIV to the others
- Being considered as a person not infected with HIV
- Not getting HIV for a second time
- No longer need to visit a doctor for HIV
- Other (please specify): __________

8. Have you ever heard of functional cure of HIV?

- Yes, and I understand what it is
- Yes, but I don’t know the details
- Never

*HIV functional cure is a status with controlled HIV replication in the absence of lifelong antiretroviral therapy after receiving some novel immunotherapy. Treatment for HIV functional cure is currently in development. Although the virus cannot be completely eliminated in a functionally cured person, a status of HIV functional cure could lead to several potential health benefits, including (i) maintaining viral load at undetectable level, (ii) restoring and stabilizing effective immune function, (iii) reducing risk of disease progression and reactivation of viruses, and (iv) having minimal risk of virus transmission.*

9. Please rate your desirability of HIV functional cure on a scale of 0 to 10. (A score of 0 represents that HIV functional cure is very undesirable, while a score of 10 represents that HIV functional cure is very desirable.)
__________________

10. If a clinical trial on HIV functional cure were offered to you in the future, how likely would you take part in it?

- Definitely yes
- Probably yes
- Maybe yes
- Maybe no
- Probably no
- Definitely no

11. Please rate the level of importance of the following factors while you are deciding to take part in a clinical trial on HIV functional cure.

|  | Very important | Moderately important | Somewhat important | A little important |
| --- | --- | --- | --- | --- |
| Safety of the therapy |  |  |  |  |
| Duration of the clinical trial |  |  |  |  |
| Incentives for participation |  |  |  |  |
| Views and support from my family and peers |  |  |  |  |
| Advice from healthcare professionals |  |  |  |  |
| Credibility of the research institution |  |  |  |  |
| Interruption of my HIV  Antiretroviral medications |  |  |  |  |

12. Please rate the level of concerns about the following situations while you are participating in a clinical trial on HIV functional cure.

|  | Very concerned | Moderately concerned | Somewhat concerned | A little concerned |
| --- | --- | --- | --- | --- |
| CD4 count going down |  |  |  |  |
| HIV viral load going up |  |  |  |  |
| Becoming infectious to the others |  |  |  |  |
| Presence of AIDS or other related complications |  |  |  |  |
| The therapy has side effects |  |  |  |  |

# Treatment-experienced participants

1. How old are you? _____ years old

2. Highest education level attained:

- Primary
- Secondary
- Post-secondary / University or above

3. What is your current socio-economic status?

- Full-time employed / self-employed
- Part-time employed / Freelancer
- Student
- Unemployed

4. In the past 1 year, have you been diagnosed with other sexually transmitted diseases?

- Yes
- No

5. In the past one year, which type of sex partners were you inclined to have sex with?

- I definitely looked for an HIV-ve sex partner
- I was inclined to find an HIV-ve sex partner
- I was inclined to find an HIV+ve sex partner
- I definitely looked for an HIV+ve sex partner
- I did not care about the HIV status of my sex partner(s)
- I did not have sex any more

6. In the past one year, have you taken any of the following recreational drugs before or during anal intercourse with your male sex partner(s)?

□ Poppers / RUSH □ Ketamine □ MDMA / Ecstasy

□ Cocaine / coke / crack □ Foxy / 0 capsule □ GHB

□ Methamphetamine / Ice □ Cannabis / Marijuana □ Meow Meow / Bath salt

□ Viagra □ Other (please specify): __________

□ I did not take any recreational drugs or I did not have anal intercourse with male in the past one year.

7. In your opinion, what are the most important impacts of an HIV cure? (Choose 3 options at most).

- No longer at risk of AIDS or HIV-related morbidity
- No longer need to take HIV medications
- Restoration and stabilization of effective immune function
- No longer transmitting HIV to the others
- Being considered as a person not infected with HIV
- Not getting HIV for a second time
- No longer need to visit a doctor for HIV
- Other (please specify): __________

8. Have you ever heard of functional cure of HIV?

- Yes, and I understand what it is
- Yes, but I don’t know the details
- Never

*HIV functional cure is a status with controlled HIV replication in the absence of lifelong antiretroviral therapy after receiving some novel immunotherapy. Treatment for HIV functional cure is currently in development. Although the virus cannot be completely eliminated in a functionally cured person, a status of HIV functional cure could lead to several potential health benefits, including (i) maintaining viral load at undetectable level, (ii) restoring and stabilizing effective immune function, (iii) reducing risk of disease progression and reactivation of viruses, and (iv) having minimal risk of virus transmission.*

9. Please rate your desirability of HIV functional cure on a scale of 0 to 10. (A score of 0 represents that HIV functional cure is very undesirable, while a score of 10 represents that HIV functional cure is very desirable.)
__________________

10. If a clinical trial on HIV functional cure were offered to you in the future, how likely would you take part in it?

- Definitely yes
- Probably yes
- Maybe yes
- Maybe no
- Probably no
- Definitely no

11. Please rate the level of importance of the following factors while you are deciding to take part in a clinical trial on HIV functional cure.

|  | Very important | Moderately important | Somewhat important | A little important |
| --- | --- | --- | --- | --- |
| Safety of the therapy |  |  |  |  |
| Duration of the clinical trial |  |  |  |  |
| Incentives for participation |  |  |  |  |
| Views and support from my family and peers |  |  |  |  |
| Advice from healthcare professionals |  |  |  |  |
| Credibility of the research institution |  |  |  |  |
| Interruption of my HIV  Antiretroviral medications |  |  |  |  |

12. Please rate the level of concerns about the following situations while you are participating in a clinical trial on HIV functional cure.

|  | Very concerned | Moderately concerned | Somewhat concerned | A little concerned |
| --- | --- | --- | --- | --- |
| CD4 count going down |  |  |  |  |
| HIV viral load going up |  |  |  |  |
| Becoming infectious to the others |  |  |  |  |
| Presence of AIDS or other related complications |  |  |  |  |
| The therapy has side effects |  |  |  |  |
