# Supplementary material for: Awareness of HIV functional cure and willingness in participating in related clinical trials: comparison between antiretroviral naïve and experienced men who have sex with men living with HIV
Source: BMC Infect Dis. 2022 Apr 15;22:383. doi: 10.1186/s12879-022-07346-x (PMC9013029; doi:10.1186/s12879-022-07346-x)
Supplement: Supplementary file 2 — Additional file 2: Table S1. Crude odds ratio for factors of importance to be considered and concerns related to participation in functional cure trial among MSM (N=356). Table S2. Crude odds ratio for important factors to be considered about and concerns related to the trial among MSM (N=356). [file 12879_2022_7346_MOESM2_ESM.docx]

**Table S1. Crude odds ratio for factors of importance to be considered and concerns related to participation in functional cure trial among MSM (N=356)**

|  | **Factors of importance** | | | | | | | **Concerns related to trial** | | | | |
| --- | --- | --- | --- | --- | --- | --- | --- | --- | --- | --- | --- | --- |
|  | **RCT1** | **RCT2** | **RCT3** | **RCT4** | **RCT5** | **RCT6** | **RCT7** | **CON1** | **CON2** | **CON3** | **CON4** | **CON5** |
| **Age group** |  |  |  |  |  |  |  |  |  |  |  |  |
| 16-29 | 1.00 | 1.00 | 1.00 | 1.00 | 1.00 | 1.00 | 1.00 | 1.00 | 1.00 | 1.00 | 1.00 | 1.00 |
| 30-39 | 0.65 | 0.86 | 0.97 | 0.88 | 0.58 | 0.41 | 0.75 | 0.93 | 0.77 | 0.71 | **0.21**** | 0.88 |
| 40-49 | 2.39 | 1.22 | 0.49 | 0.73 | 1.01 | **0.38*** | **0.42*** | 0.89 | 0.62 | 0.67 | **0.25*** | 0.99 |
| 50 or over | 1.10 | 1.69 | 0.82 | 1.23 | 0.79 | 1.03 | 3.11 | 1.17 | 0.76 | 0.61 | **0.18**** | 1.24 |
| **Education level** |  |  |  |  |  |  |  |  |  |  |  |  |
| Secondary or below | 1.00 | 1.00 | 1.00 | 1.00 | 1.00 | 1.00 | 1.00 | 1.00 | 1.00 | 1.00 | 1.00 | 1.00 |
| Post-secondary or above | 1.79 | 0.71 | 0.82 | **0.43***** | 0.58 | **2.11*** | 1.20 | 1.23 | 1.37 | 1.52 | **2.08*** | 1.11 |
| **STD history in the past year** | 4.12 | 0.73 | 1.33 | 0.83 | 1.03 | 0.88 | 1.75 | 0.84 | 0.93 | 1.42 | 0.72 | 0.61 |
| **Sexual activity after HIV diagnosis** | 0.92 | 0.92 | 0.75 | **0.59*** | 1.22 | 1.25 | 0.58 | **0.54*** | 0.70 | 0.95 | **0.46*** | 0.63 |
| **Engagement in chemsex** | 0.65 | 1.20 | 1.32 | 0.84 | 1.11 | 0.65 | 0.85 | 1.04 | 0.70 | 0.86 | 0.73 | 0.60 |
| **CD4 cell count (cells/uL)** |  |  |  |  |  |  |  |  |  |  |  |  |
| ≥ 500 | 1.00 | 1.00 | 1.00 | 1.00 | 1.00 | 1.00 | 1.00 | 1.00 | 1.00 | 1.00 | 1.00 | 1.00 |
| < 500 | 0.34 | 0.81 | 1.21 | 1.36 | 0.83 | 0.94 | 0.83 | 1.23 | 1.43 | 1.00 | **2.02*** | 1.32 |
| **About functional cure** |  |  |  |  |  |  |  |  |  |  |  |  |
| Anticipation | 2.81 | 1.51 | 1.46 | 1.12 | 1.37 | 1.84 | 1.32 | 1.27 | 1.17 | 1.12 | 1.01 | 1.27 |
| Awareness | 0.60 | 0.84 | 1.26 | 1.27 | 0.88 | 0.88 | 1.08 | 0.70 | 1.31 | 1.25 | 0.77 | **0.54*** |
| Willingness | 3.09 | 0.73 | 0.43 | 0.46 | **4.64**** | 2.57 | 1.61 | 0.90 | 0.68 | 0.64 | 0.88 | 1.12 |

* p<0.05 ** p<0.01 *** p<0.001

MSM men who have sex with men

| **RCT1** | Safety of the therapy | **RCT5** | Advice from healthcare professionals | **CON1** | CD4 count going down |
| --- | --- | --- | --- | --- | --- |
| **RCT2** | Duration of the clinical trial | **RCT6** | Credibility of the research institution | **CON2** | HIV viral load going up |
| **RCT3** | Incentives for participation | **RCT7** | Interruption of my HIV antiretroviral medications | **CON3** | Becoming infectious to the others |
| **RCT4** | Views and support from my family and peers |  |  | **CON4** | Presence of AIDS or other related complications |
|  |  |  |  | **CON5** | The therapy has side effects |

**Table S2. Crude odds ratio for important factors to be considered about and concerns related to the trial among MSM (N=356)**

|  | **Important factors to be considered about the trial** | | | | | | | **Concerns related to the trial** | | | | |  |
| --- | --- | --- | --- | --- | --- | --- | --- | --- | --- | --- | --- | --- | --- |
|  | **RCT1** | **RCT2** | **RCT3** | **RCT4** | **RCT5** | **RCT6** | **RCT7** | **CON1** | **CON2** | **CON3** | **CON4** | **CON5** | |
| **IMP1** | 0.97 | 1.46 | 1.17 | 0.70 | 0.79 | 1.09 | 1.74 | 1.05 | 0.92 | 1.11 | 1.69 | 1.18 | |
| **IMP2** | 0.97 | 1.10 | 1.21 | 1.22 | 0.70 | 1.05 | 1.02 | **2.03**** | 1.25 | 0.99 | 1.31 | 1.60 | |
| **IMP3** | 1.75 | 2.15 | **2.09*** | 1.56 | 0.77 | 0.85 | 3.11 | 0.94 | 1.61 | 1.16 | 0.99 | 0.82 | |
| **IMP4** | 3.85 | 0.88 | 0.93 | 1.11 | 0.99 | 1.22 | 1.26 | 0.85 | 0.80 | 1.12 | 0.77 | 1.18 | |
| **IMP5** | 1.80 | **0.34***** | **0.57*** | 0.64 | 0.89 | 0.63 | **0.40**** | 1.41 | 1.22 | 1.05 | 1.50 | 1.28 | |
| **IMP6** | 1.59 | 1.14 | 1.03 | 1.42 | 0.77 | 0.78 | 1.32 | 0.76 | 1.07 | 1.17 | 0.96 | 0.78 | |
| **IMP7** | 1.63 | 0.55* | **0.38**** | **0.36**** | 0.62 | 0.88 | **0.49*** | 1.27 | 1.46 | 1.48 | 1.38 | 1.12 | |
| **RCT1** | -- | -- | -- | -- | -- | -- | -- | **14.21***** | **26.91***** | **6.21**** | **5.95**** | **22.98***** | |
| **RCT2** | -- | -- | -- | -- | -- | -- | -- | 1.13 | 1.16 | 1.61 | 1.23 | **2.67**** | |
| **RCT3** | -- | -- | -- | -- | -- | -- | -- | 0.91 | 0.67 | 1.18 | 0.52 | 0.97 | |
| **RCT4** | -- | -- | -- | -- | -- | -- | -- | 1.17 | 0.80 | 1.05 | 0.60 | 0.86 | |
| **RCT5** | -- | -- | -- | -- | -- | -- | -- | 1.64 | 2.14 | 1.48 | 1.44 | 1.36 | |
| **RCT6** | -- | -- | -- | -- | -- | -- | -- | 1.71 | **2.30*** | 1.19 | 1.96 | 1.45 | |
| **RCT7** | -- | -- | -- | -- | -- | -- | -- | **2.30**** | **2.81**** | **2.01*** | 2.02 | 1.85 | |

* p<0.05 ** p<0.01 *** p<0.001
MSM men who have sex with men

| **IMP1** | No longer need to take HIV medications | **RCT1** | Safety of the therapy | **CON1** | CD4 count going down |
| --- | --- | --- | --- | --- | --- |
| **IMP2** | Restoration and stabilization of effective immune function | **RCT2** | Duration of the clinical trial | **CON2** | HIV viral load going up |
| **IMP3** | Not getting HIV for a second time | **RCT3** | Incentives for participation | **CON3** | Becoming infectious to the others |
| **IMP4** | No longer need to visit a doctor for HIV | **RCT4** | Views and support from my family and peers | **CON4** | Presence of AIDS or other related complications |
| **IMP5** | No longer at risk of AIDS or HIV-related morbidity | **RCT5** | Advice from healthcare professionals | **CON5** | The therapy has side effects |
| **IMP6** | No longer transmitting HIV to the others | **RCT6** | Credibility of the research institution |  |  |
| **IMP7** | Being considered as a person not infected with HIV | **RCT7** | Interruption of my HIV antiretroviral medications |  |  |
